# Protein energy malnutrition increases arginase activity in monocytes and macrophages

**DOI:** 10.1186/1743-7075-11-51

**Published:** 2014-10-24

**Authors:** Karina Corware, Vanessa Yardley, Christopher Mack, Steffen Schuster, Hafid Al-Hassi, Shanthi Herath, Philip Bergin, Manuel Modolell, Markus Munder, Ingrid Müller, Pascale Kropf

**Affiliations:** Department of Medicine, Section of Immunology, Faculty of Medicine, Imperial College London, Norfolk Place, London, W2 1PG UK; Immunology and Infection Department, London School of Hygiene and Tropical Medicine, London, UK; Department of Biochemistry, WHO Immunology Research and Training Center, University of Lausanne, Lausanne, Switzerland; School of Biological Sciences, Royal Holloway, University of London, Egham, UK; International AIDS Vaccine Initiative Human Immunology Laboratory, Faculty of Medicine, Imperial College London, London, UK; Department of Cellular Immunology, Max-Planck-Institute for Immunobiology and Epigenetics, Freiburg, Germany; Third Department of Medicine (Hematology, Oncology, and Pneumology), University Medical Center Mainz, Mainz, Germany

**Keywords:** Arginase, Macrophages, Monocytes, Nitric oxide, Leishmaniasis

## Abstract

**Background:**

Protein energy malnutrition is commonly associated with immune dysfunctions and is a major factor in susceptibility to infectious diseases.

**Methods:**

In this study, we evaluated the impact of protein energy malnutrition on the capacity of monocytes and macrophages to upregulate arginase, an enzyme associated with immunosuppression and increased pathogen replication.

**Results:**

Our results show that monocytes and macrophages are significantly increased in the bone marrow and blood of mice fed on a protein low diet. No alteration in the capacity of bone marrow derived macrophages isolated from malnourished mice to phagocytose particles, to produce the microbicidal molecule nitric oxide and to kill intracellular *Leishmania* parasites was detected. However, macrophages and monocytes from malnourished mice express significantly more arginase both *in vitro* and *in vivo*. Using an experimental model of visceral leishmaniasis, we show that following protein energy malnutrition, the increased parasite burden measured in the spleen of these mice coincided with increased arginase activity and that macrophages provide a more permissive environment for parasite growth.

**Conclusions:**

Taken together, these results identify a novel mechanism in protein energy malnutrition that might contributes to increased susceptibility to infectious diseases by upregulating arginase activity in myeloid cells.

**Electronic supplementary material:**

The online version of this article (doi:10.1186/1743-7075-11-51) contains supplementary material, which is available to authorized users.

## Background

Undernutrition results from insufficient dietary intake, poor absorption and/or inadequate use of the nutrients consumed. It is a major health concern, mainly throughout the developing world and children are especially affected. An estimated 826 million people in the world are undernourished, of those 95.9% are in the developing world [1]; 178 million children under the age of 5 are stunted and 55 million are wasted [2]. In 2011, 6.9 million children under 5 years died, one third of these deaths were related to increased susceptibility to infections due to undernutrition [2]. Protein energy malnutrition (PEM) is thought to be one of the major causes of immunodeficiency. PEM and infection have always been closely associated, as PEM is a common cause of susceptibility to infections. Undernutrition in children negatively impacts on thymic development, therefore compromising immune responses [3]. In malnourished patients, both innate and acquired immunity are affected [4, 5] and common immune defects are an imbalance in the ratio of CD4/CD8^+^ T cells [6], low expression levels of CD69 on lymphocytes [7], biased T helper cell responses [8], reduced antibody responses [6]; impaired phagocytosis by macrophages [9, 10], lower nitrite/nitrate concentrations in wound fluid [11] and decreased NF-kappaB activation by macrophages [12] have also been shown in experimental models of PEM.

Arginase, an enzyme that catalyses the conversion of L-arginine, can be upregulated in murine macrophages, dendritic cells and neutrophils [13], however, human neutrophils constitutively express arginase (reviewed in [13]). There are two isoforms of arginase in mammals, arginase I and arginase II [14, 15]. The catabolism of L-arginine by arginase can regulate the availability of L-arginine and therefore the efficiency of T cell responses: increased catabolism of L-arginine by arginase results in the depletion of L-arginine from the microenvironment; since L-arginine is essential for efficient T cell activation, this decrease in L-arginine results in impaired T cell responses [13, 16–18]. In addition, the catabolism of L-arginine by arginase results in the production of ornithine, which is further catabolised into polyamines that are crucial for cell division; and into proline, which is the building block for collagen synthesis. L-arginine is also the substrate for nitric oxide synthase (NOS), that catabolises L-arginine into nitric oxide (NO), a molecule critical for the regulation of vascular homeostasis, neurotransmission and the killing of many pathogens [19]. Therefore, by competing for the shared substrate L-arginine, increased arginase activity also modulates the production of NO. Consequently, deficiencies in L-arginine metabolism can disrupt many cellular and organ functions.

Increased arginase activity is common to several pathological and physiological conditions: it has been prominently described in cancer [16, 18], but also in asthma, myocardial infarction, pregnancy and infectious and autoimmune diseases (summarised in [13]). We have recently shown that increased arginase activity is a marker of disease severity in HIV seropositive (HIV+) patients [20], in patients with visceral leishmaniasis and HIV co-infection [21] and in patients with visceral [22] and cutaneous [23] leishmaniasis. Malnutrition is a major contributor to both progression and severity of visceral leishmaniasis (VL) and several studies in the mouse have shown that PEM and micronutrient deficient diet exacerbates VL [24].

Nutrition has been shown to impact on the phenotype of macrophages: in obesity, adipose tissue is characterized by infiltration of inflammatory macrophages, in contrast to lean adipose tissue, where a majority of anti-inflammatory macrophages are present [25]. In the present study, we tested the hypothesis that malnutrition impacts on macrophage effector functions and results in increased arginase activity, therefore contributing to increased susceptibility to disease.

## Methods

### Mice

Specific pathogen free female BALB/c mice (18 g) were purchased from Charles River (UK) and were kept in individually vented cages. The animal colonies were screened regularly for mouse pathogens and consistently tested negative.

#### Ethics statement

Animal experiments were performed in accordance with home office and institutional guidelines. The project licence (PPL 70/6712) was approved by the Imperial College Central Animal Welfare & review Body (AWERB) committee.

Mice were fed on the different diets following a 3-day period of acclimatisation. Control mice were fed a diet consisting of 14.4% protein, 61.7% carbohydrates and 2.7% fat (RM1, Harlem Laboratories Inc, Madison, Additional file 1). The group of mice that was malnourished (MN group) was fed a diet consisting of 0.7% protein, 87.0% carbohydrates and 4.1% fat (Modification of TestDiet AIN-93 M w/No protein, TestDiet, Additional file 2). Both diets had similar amounts of calories: control group =3.5 kcal/g and MN group: 3.85 kcal/g and both groups of mice had *ad libitum* access to food and water.

### Sample collection

Bone marrow cells were obtained by flushing the femurs of BALB/c mice and precursor cells were used immediately for flow cytometry or were frozen in PBS containing 1% protease inhibitor cocktail (Sigma) for the determination of arginase activity. To obtain bone marrow derived macrophages (BMMΦ), fresh precursor cells were cultured for 8 days in a humid atmosphere at 37°C and 10% CO_2_ in hydrophobic Teflon bags in DMEM (Sigma) containing 10% heat-inactivated FCS (Gibco), 5% horse serum (Gibco), the supernatant of L929 fibroblasts at a final concentration of 15% (v/v) as a source of colony stimulating factor (CSF), 50 IU/ml penicillin, 50 μg/ml streptomycin, and 292 μg/ml L-glutamine (Sigma) as described in [26]. To obtain bone marrow-derived neutrophils, fresh precursor cells were cultured for 7 days as described in [27].

Peripheral blood mononuclear cells (PBMCs): blood was collected in EDTA tubes by cardiac puncture and PBMCs were isolated by density gradient centrifugation on Histopaque-1077 (Sigma). Before harvesting the interphase, the plasma was collected and frozen for further analysis. Cells were washed in phosphate buffered saline (PBS) and were used immediately for flow cytometry or were frozen in PBS containing 1% protease inhibitor cocktail (Sigma) for the determination of arginase activity.

Spleen cells: spleens from BALB/c mice were homogenised in PBS and red cells were lysed with red cell lysis buffer as described in [27] and were frozen in PBS containing 1% protease inhibitor cocktail (Sigma) for the determination of arginase activity.

### Macrophage activation

Mature BMMΦ and mature BM-derived neutrophils were harvested and 5 × 10^5^ BMMΦ/ml were plated in DMEM (Sigma) containing 5% heat-inactivated FCS (Gibco), 50 IU/ml penicillin, 50 μg/ml streptomycin, and 292 μg/ml L-glutamine (Sigma) and 5 × 10^-5^ M 2-mercaptoethanol (Sigma). BMMΦ were stimulated with 20U/ml IL-4 or with 100U/ml IFN-γ and 500U/ml TNF-α (Peprotech) or with 1 μg LPS (Sigma) for 72 hours, unstimulated macrophages were used as control. Mature BM-derived neutrophils were stimulated with 20U/ml IL-4 (Peprotech) and unstimulated mature BM-derived neutrophils were used as control.

### Phagocytosis assay

Mature BMMΦ (1 × 10^5^ BMMΦ) were incubated for 1 hour with pHrodo™ Bioparticles® fluorescent particles according to the manufacturer’s protocol (Molecular Probes). Analysis of the mean fluorescence intensities (MFI) was performed on a LSRII (BD Bioscience) and results were analyzed using Summit v4.3 software.

### Flow cytometry

Antibodies used were as follows: anti-Ly-6C (clone HK1.4, eBioscience), anti-Ly-6G (clone RB6-8C5, eBioscience), anti-F4/80 (clone BM8, BD Biosciences), anti-arginase I (Polyclonal sheep IgG, R&D). Cells were washed with PBS, the fixation step was performed with 2% formaldehyde in PBS and the permeabilisation step with 0.5% saponin in PBS. Analysis was performed on a LSRII (BD Bioscience) and results were analyzed using Summit v4.3 software.

The percentages of positive cells with the isotype controls were <1%.

### Determination of nitrite and arginase activity

NO_2_- accumulated in the supernatant was used as an indicator of NO production and measured using the Griess reagent as described in [28]. Culture supernatants were collected after 48 h, and equal volumes of macrophage culture supernatants and Griess reagent (1% sulphanilamide/0.1% N-(1-naphthyl)ethylenediamine dihydrochloride/2.5% H_3_PO_4_) were mixed and incubated for 10 min at room temperature. Absorbance was measured at 540 nm. Nitrite concentration was determined using NaNO_2_ as standard.

The enzymatic activity of arginase was measured as previously described [29]. Briefly, the cell lysate was activated by heating for 10 min at 56°C. L-arginine hydrolysis was conducted by incubating the activated lysate with 0.5 M L-arginine (pH 9.7) at 37°C for 15 to 120 minutes. The reaction was stopped with H_2_SO_4_(96%)/H_3_PO_4_(85%)/H_2_O (1:3:7, v/v/v, VWR). α-isonitrosopropiophenone (ISPF, dissolved in 100% ethanol, Sigma) was added and incubated for 45 min at 100°C, followed by 30 min at 4°C. A standard curve was obtained by treating serially diluted urea with ISPF and incubated in the final step. The optical density (OD) was measured at 550 nm.

Protein concentration of samples was measured using the BCA Protein Assay kit (Pierce) as previously described [29]. One unit of enzyme activity is defined as the amount of enzyme that catalyzes the formation of 1 μmol of urea per min.

### Determination of L-arginine and L-ornithine concentrations

The concentration of L-arginine and L-ornithine in plasma were measured by ion exchange chromatography and quantified by post-column ninhydrin derivatisation using an amino acid analyser (AminoTac JLC- 500/V) as described previously [30].

### Infection of mice and macrophages with *Leishmania infantum*

#### Mice

*Leishmania* (*L*) *infantum* amastigotes (MHOM/MA/67/ITMPA263 (MON-1) were derived from infected RAG1.B6 mice. BALB/c mice were infected 7 days after start of the experiment with 3 × 10^6^ amastigotes in the lateral tail vein and were culled 9 days later. Spleen cells were isolated as described above and 5 × 10^6^ cells were resuspended in 1 ml M199 containing 50 IU/ml penicillin, 50 μg/ml streptomycin, and 292 μg/ml L-glutamine (Sigma), 2% hepes and 4 μM sodium bicarbonate (Sigma) and 10% fetal bovine serum (Gibco) and incubated in a humid atmosphere at 26°C and 5% CO_2_. Eight days later, the wells were resuspended carefully and the transformed promastigotes were fixed with PBS containing 2% formaldehyde and counted using a haemocytometer.

#### Macrophages

5 × 10^5^ BMMΦ/ml cells were plated and stimulated with 20U/ml IL-4 or with 100U/ml IFN-γ and 500U/ml TNF-α (Peprotech). Unstimulated macrophages were used as control. Four hours later, the cultures were infected with 25 × 10^5^/ml *L. infantum* promastigotes. After 96 h, the macrophages were washed and lysed with HEPES-buffered medium containing 0.008% SDS [31] and 1 ml of complete M199 was added to each well, incubated and the transformed promastigotes were counted as described above.

### Statistical analyses

Data were evaluated for statistical differences using a two-tailed Mann–Whitney test (GraphPad Prism 5) and differences were considered statistically significant at *p* <0.05. Results are expressed as mean with 95% CI.

## Results

### Impact of protein energy malnutrition on weight loss

**Figure 1:**
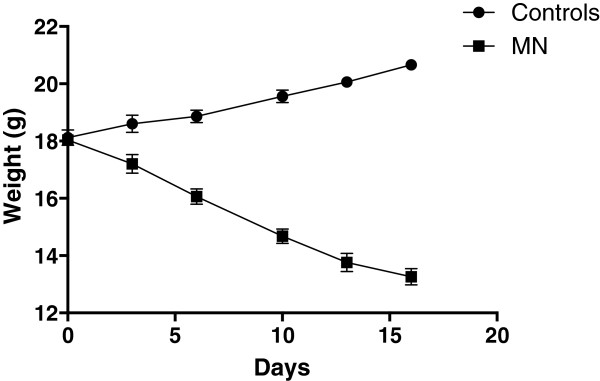
**Weight loss in mice fed on a low protein diet.** Groups of BALB/c (n =5) mice were fed with a low protein diet (=malnourished (MN) group, 0.7% protein) or normal diet (=control group, 14.4% protein) for 16 days. Their weight was measured at regular intervals. Data show the results of one representative experiment out of five independent experiments. Results are expressed as mean with 95% CI.

We first set up a model of malnutrition in mice to test the impact of PEM, using an isocaloric diet containing 0.7% protein. As shown in Figure 1, mice fed on the low protein diet lost weight steadily and on day 16, the mice had lost an average of 26.5% of their initial weight, whereas the control mice fed on a 13.8% diet gained weight. Mice fed on the low calorie diet ate 2.53 g/mouse/day and mice fed on a 13.8% diet ate 3.01 g /mouse/day.

### Impact of malnutrition on the frequency of monocytes in the bone marrow and peripheral blood mononuclear cells

**Figure 2:**
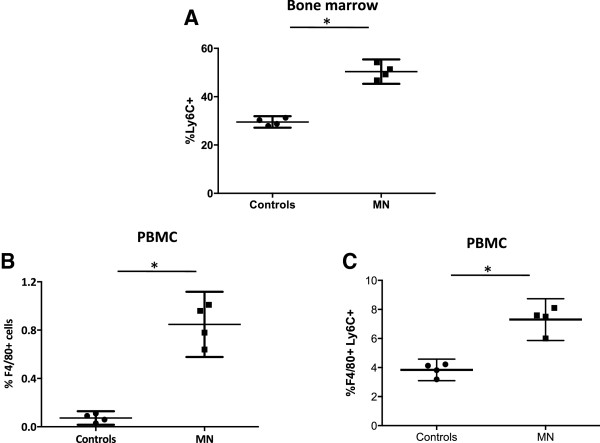
**Percentages of monocytes in the bone marrow and peripheral blood mononuclear cells.** The percentages of Ly6C + cells in BM **(A)**, F4/80+ **(B)** and F4/80 + Ly6C + cells **(C)** in the PBMCs were determined by flow cytometry in cells isolated from mice fed on a low protein (MN, n =4) or normal (control, n =4) diet for 16 days. Data show the results of one representative experiment out of three independent experiments. *: *p* <0.05. Results are expressed as mean with 95% CI.

Next we studied the impact of malnutrition on monocytes. Since Ly6C + bone marrow monocytes are released into the peripheral blood [32], we assessed their frequency in bone marrow (BM) and peripheral blood by flow cytometry. Results presented in Figure 2A show a significant increase in the frequency of Ly6C^high^ monocytes in the BM isolated from malnourished (MN) mice (p =0.0286). We then measured the frequency of F4/80+ monocytes in the PBMCs from both groups of mice and detected a significant increase in the frequency of F4/80+ monocytes in the MN group (*p* = 0.0286, Figure 2B). As previously described [33], the majority (>91%, data not shown) of the blood monocytes express low levels of Ly6C. Interestingly, the frequency of inflammatory F4/80 + Ly6C + cells was significantly higher in the blood of MN mice (*p* =0.0286, Figure 2C).

These results show that in MN mice, the frequencies of Ly6C + monocytes and F4/80+ monocytes are increased in BM and PBMCs, respectively.

### Impact of malnutrition on macrophage effector functions *in vitro*

**Figure 3:**
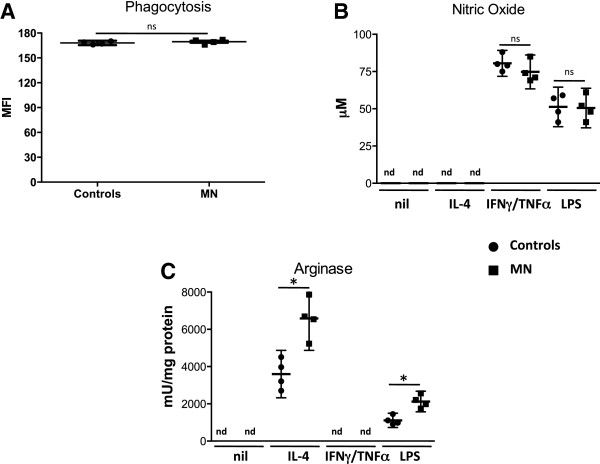
**Bone marrow derived macrophage effector functions.** Bone marrow cells were isolated from the bone marrow from mice fed on a low protein (MN, n =4) or normal (control, n =4) diet for 16 day and were differentiated in conditioning medium. Eight days later, the capacity of these cells to phagocytose particles was determined by flow cytometry **(A)**. In addition, BMMΦ were activated with IL-4, IFN-γ and TNF-α, LPS or left unactivated (nil) and the production of nitric oxide **(B)** and the activity of arginase **(C)** were measured as described in material and methods. Data show the results of one representative experiment out of four independent experiments. *: *p* <0.05. Box = interquartile range and median; whiskers = range. nd = not detectable.

Next we differentiated bone marrow cells from both groups of mice into mature BMMΦ and assessed their effector functions. Following 8 days of differentiation in conditioning medium, the number of F4/80+ cells was similar in both groups (*p* =0.4857, data not illustrated). We measured the capacity of these cells to phagocytose BioParticles and the results in Figure 3A show that BMMΦ derived from control and MN mice have a similar capacity to phagocytose, as the MFIs of the internalised fluorescent particles were comparable (*p* =0.4857). We then compared the capacity of BMMΦ derived from control and MN mice to produce nitric oxide (NO) and upregulate arginase. As shown in Figure 3B, BMMΦ from both groups of mice produce similar levels of NO when stimulated with IFN-γ and TNF-α (*p* =0.2000) or with LPS (*p* =0.8939). Unstimulated BMMΦ and BMMΦ stimulated with IL-4 did not produce detectable levels of NO (Figure 3B). Next, we measured the capacity of BMMΦ to upregulate arginase; results presented in Figure 3C show that in response to IL-4, BMMΦ derived from MN mice express significantly increased arginase activity (*p* =0.0286). Similarly, following stimulation with LPS, arginase activity was significantly higher in BMMΦ derived from MN mice (*p* =0.0286, Figure 3C). The capacity of bone marrow-derived neutrophils to upregulate arginase in response to IL-4 was similar in both groups (26.3 vs 28.53 mU/mg protein, *p* =0.4857).

These results show that whereas the production of NO is similar in both groups of mice, activation of BMMΦ derived from MN mice with IL-4 or LPS results in increased arginase activity.

### Impact of malnutrition on macrophage effector functions *in vivo*

**Figure 4:**
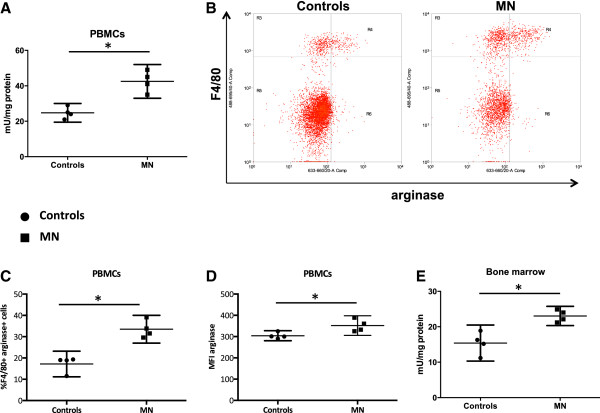
**Arginase activity ex vivo** ***.***Arginase activity was measured by enzymatic assay in PBMCs **(A)** and bone marrow cells ex vivo **(E)**. The phenotype **(B)** and percentage **(C)** of arginase expressing and the MFI of arginase **(D)** was determined by flow cytometry. Bone marrow cells were isolated from 4 mice and pooled, and PBMCs were isolated from the peripheral blood from 4 mice and pooled. Data show the results of one representative experiment out of four independent experiments. *: *p* <0.05. Box = interquartile range and median; whiskers = range.

The results presented in Figure 3C show that BMMΦ from MN mice have the capacity to express higher arginase activity than those from control mice. To determine whether arginase was also increased *in vivo* in mice fed on a low protein diet, we measured the levels of arginase activity in PBMCs. As shown in Figure 4A, the levels of arginase activity were significantly higher in PBMCs from malnourished mice (*p* =0.0298). Next the phenotype of arginase + cells was identified by flow cytometry. As shown in Figures 4B, the large majority of arginase-1 positive cells are F4/80+ in the PBMCs, and we have previously showed that arginase 1, but not arginase 2, is upregulated in F4/80+ cells [26]. These cells were Ly6G negative, therefore excluding neutrophils (data not shown), In the PBMCs from MN mice, the percentage of F4/80 + arginase + cells (*p* =0.0286, Figure 4C) and the MFI of arginase (*p* =0.0421, Figure 4D) were also significantly increased as compared to controls. Next, we determined whether increased levels of arginase activity in the PBMCs coincide with lower levels of L-arginine [30]: our results show that the levels of L-arginine are significantly lower in the plasma of MN mice (65.5 μM vs controls: 160.3 μM, *p* =0.0286, data not illustrated). In agreement with these results, the levels of L-ornithine, one of the products of arginase-mediated L-arginine catabolism, were increased (MN: 121.3 vs controls: 89.5*, p* =0.0286, data not illustrated).

Arginase activity was also significantly higher in the bone marrow (*p* =0.0286, Figure 4E), but the phenotype of arginase + cells was not identified because the frequencies of arginase + cells were too low.

### Impact of malnutrition on disease development in *Leishmania infantum*-infected mice

**Figure 5:**
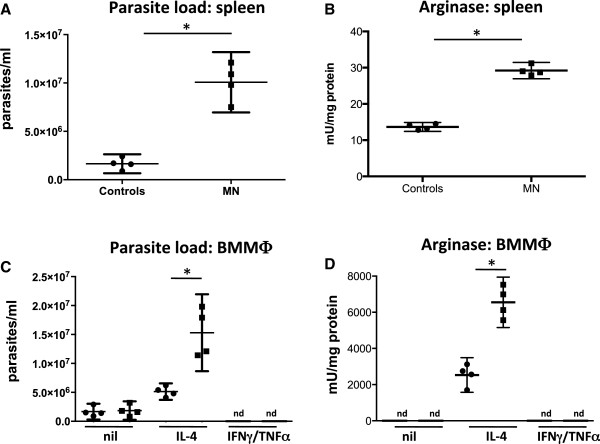
**Impact of malnutrition on parasite replication and arginase activity in** ***Leishmania infantum*** **-infected BALB/c mice.** Groups of BALB/c (n =5) mice were fed with a low protein diet (=malnourished (MN) group, 0.7% protein) or normal diet (=control group, 14.4% protein) and 7 days later, were infected with *L. infantum*. After nine days, parasite load **(A)** and arginase activity were measured in the spleens **(B)**. BMMΦ were activated with IL-4, IFN-γ and TNF-α or left unactivated (nil) and were infected with *L. infantum* for 4 days and parasite replication **(C)** and arginase activity **(D)** were determined as described in material and methods. Data show the results of one representative experiment out of two independent experiments. *: *p* <0.05. nd = not detectable.

We have previously showed that increased arginase correlates with increased parasite replication and that competitive inhibition of arginase resulted in reduced parasite growth [26, 30]. Here we determined whether the increased arginase activities observed in mice fed with a low protein diet did coincide with increased parasite load. Seven days after the start of the diets, mice were infected i.v. with *Leishmania* (*L*)*. infantum* and 8 days later, parasite load and arginase activities were measured in the spleen. As shown in Figure 5A, the increased parasite load in the spleen of MN mice (*p* =0.0286) coincided with increased arginase activity (*p* =0.0286, Figure 5B).

In macrophages, efficient parasite replication is controlled at least in part by arginase: catabolism of L-arginine by arginase results in the production of polyamines that the parasites use for their replication; competitive inhibition of arginase reduced the production of polyamines in BMMΦ activated with IL-4 and thereby clearly reduced parasite replication [26]. Since BMMΦ derived from MN mice express more arginase, we assessed whether this provides a more permissive environment for parasite growth; we activated BMMΦ with IL-4 and infected them with *L. infantum* and measured their growth as well as arginase activity; unstimulated macrophages as well as macrophages stimulated with IFN-γ and TNF-α were used as controls. As shown in Figure 5C, *L. infantum* grew significantly better in IL-4-stimulated BMMΦ derived from MN mice (*p* =0.0286), and this increase in parasite burden coincided with increased arginase activity (*p* =0.0286, Figure 5D). As shown previously [26], no parasites could be detected in BMMΦ activated with IFN-γ and TNF-α; no significant difference was observed in parasite growth in unstimulated BMMΦ derived from MN and control mice (*p* =0.6857, Figure 5C).

The results presented in Figure 5 show that *L. infantum*-infected mice on a low protein diet have a significantly higher parasite burden paralleled by significantly increased arginase activity, which provides a more permissive environment in host macrophages for parasite growth.

## Discussion

PEM is one of the major causes of immunosuppression, modifying both innate and adaptive immunity. It is highly prevalent in low-income countries, particularly in children [4]. In the developed world, PEM is also a serious health concern, mainly seen in the elderly, with up to 10% of older people undernourished in nursing homes and up to 50% of older people undernourished when discharged from hospital [34]. Malnutrition is associated with a plethora of problems, such as fatigue, anaemia, cognitive abnormalities, immune dysfunctions and increased susceptibility to infections [34]. Monocytes and macrophages play a major role in the elimination of pathogens. Here we show that the frequency of monocytes is increased in the bone marrow and PBMCs of MN mice, suggesting a dysregulation in the haematopoiesis in these mice. This is in agreement with a study by Borelli et al. [35] that showed a 2.5-fold increase in the number of peripheral blood monocytes. Whereas macrophages and monocytes can be instructed to kill intracellular pathogens, they can also act as “safe target”. Indeed, in *Leishmania major* infected nonhealing mice, macrophage-granulocytes precursor cells were shown to be dramatically increased during infection, thereby offering a large number of host cells for the parasites to infect [36, 37]. Since these cells have not yet developed their full microbicidal potential [37], the parasites cannot be killed efficiently and the myeloid cells represent therefore a more permissive environment for the parasites to survive. We also showed an increase in the frequency of F4/80 + Ly6C + inflammatory macrophages in the blood of MN mice; these macrophages have been shown to be rapidly recruited to sites of inflammation. Since malnutrition has been associated with chronic inflammation [38], this might explain the increased frequency of these macrophages during PEM.

Whereas we detected arginase activity in the BM cells, we were not able to able to identify the phenotype of arginase-expressing cells. Since other cells than macrophages have been shown to express arginase, it is possible that cells such as dendritic cells, neutrophils [27, 28] or innate lymphoid cells [39] might contribute.

Here, we showed that PEM did not impact on the capacity of BMMΦ to produce NO in response to activation with IFN-γ and TNF-α or LPS; nor on the capacity of BMMΦ capacity to kill intracellular *Leishmania* parasites. We also showed that *in vitro*, bone marrow cells from malnourished mice did proliferate and differentiate similarly to those from control mice as both the number of cells after 8 days of differentiation were similar. Furthermore, phagocytosis of bioparticles was not impaired in BMMΦ derived from malnourished mice. In contrast, it has been previously shown that some macrophage functions are altered during PEM: the phagocytic capacity and production of superoxide anions, indicative of their microbicidal competence, were shown to be impaired [10, 40, 41], suggesting that increased susceptibility to disease might be due to an impaired capacity of phagocytic cells to kill pathogens. The discrepancies between these studies and ours could be due to the different macrophages used: BMMΦ in our study versus elicited peritoneal exudate macrophages in the other studies [10, 40, 41]. Macrophages derived from different compartments such as bone marrow, spleen or peritoneum have different capacities to phagocytose dextran beads [42]. It is also possible that the different diets used, characterised by differences in calories, fat, micronutrient and protein contents, also impact on macrophage effector functions.

We have previously shown that bone marrow derived macrophages from younger mice express significantly higher levels of arginase 1 than aged mice and promote parasite growth more efficiently [43]. Furthermore, *L. major*-infected younger mice develop exacerbated lesion pathology and higher parasite burdens than aged mice [43]. A previous study from 1984 showed that arginase was decreased in the saliva of children with different grades of PEM [44]. We have previously compared the levels of arginase activity in the saliva of severely malnourished patients with visceral leishmaniasis and severely malnourished patients with visceral leishmaniasis and HIV co-infection and did not find significant differences or correlation between the levels of malnutrition and the levels of arginase activity [21]; nor did we find differences when compared to healthy controls (P. Kropf, unpublished data).

Our results show that BMMΦ derived from malnourished mice express significantly more arginase in response to IL-4 or LPS, and we also measured considerably more arginase activity in the bone marrow cells and PBMCs of malnourished mice directly *ex vivo*. We and others have shown that increased arginase activity is associated with the severity of a variety of infectious diseases [20, 21, 23, 45, 46]. At least two distinct arginase-mediated mechanisms can impact on the severity of disease:

i) Increased catabolism of L-arginine by arginase results in the depletion of L-arginine from the microenvironment; since L-arginine is essential for efficient T cell activation, this decrease in L-arginine results in impaired T cell responses [17, 18, 47, 48]. In an experimental model of leishmaniasis, we have recently shown that increased arginase activity causes local depletion of L-arginine, which impairs the capacity of T cells in the lesion to proliferate and to produce interferon-γ [30]. Healing, induced by chemotherapy, resulted in control of arginase activity and reversal of local immunosuppression; competitive inhibition of arginase as well as supplementation with L-arginine restored T cell effector functions and reduced pathology and parasite growth at the site of lesions [30]. Indeed, our results also show that the levels of L-arginine are lower in MN mice. L-arginine is derived from 1) turnover of cellular protein; 2) from endogenous synthesis: by *de novo* synthesis from citrulline; and 3) the diet via protein, which contained from 3-15% of L-arginine [14, 15]. It is therefore likely that insufficient level of protein in the diet result in reduced levels of L-arginine in plasma [49–51]. Consequently, both PEM and increased levels of arginase might contribute to the lower level of L-arginine.

PEM malnutrition not only results in lower levels of L-arginine, it also results in severe alteration of the levels of different amino acid [49–51]. It is likely that these changes impact on macrophage effector functions; for example, glutamine deprivation impacts on antigen presentation, HLA-DR expression and cytotoxic effect of TNF-α of macrophages [52]. Furthermore, increased intracellular levels of arginase-induced L-arginine catabolites such as ornithine, putrescine, spermine and spermidine can downregulate macrophage [53–55]. Of note, we have previously shown that *in vitro*, L-arginine deprivation did not impact on arginase activity and NO production [56].

ii) Increased arginase activity has also been shown to promote the growth of *Leishmania* parasites directly via the polyamine synthesis in macrophages [26]: arginase hydrolyses L-arginine to urea and ornithine; the latter is the main intracellular source for the synthesis of polyamines, which are necessary for parasite growth. Inhibition of arginase in activated BMMΦ clearly reduced parasite replication [26]. Indeed, our results show that BMMΦ derived from MN mice express significantly more arginase and provide a more permissive environment for *Leishmania* replication. Furthermore, the increased parasite burden in the spleen of MN BALB/c mice infected with *L. infantum* coincides with increased arginase activity.

To the best of our knowledge, this is the first study that shows that PEM results in increased arginase activity in monocytes and macrophages, however the mechanisms responsible for this upregulation of arginase have not been identified yet. Upregulation of arginase has been extensively described in murine macrophages: cytokines such as IL-4 and IL-13, which can synergize with IL-10 and IL-21, as well as IL-6, induce the expression of arginase; in addition, lipospolysaccharide and lipoprotein, as well as inflammatory stimuli such as thioglycollate, carrageenan and casein can also induce arginase [13]. Therefore, it is tempting to speculate that upregulation of arginase is induced as a result of PEM-induced inflammation. Whereas arginase upregulation in macrophages during PEM is STAT6- or TLRs- dependent remains to be established [57].

## Conclusion

Our results show that macrophages and monocytes from malnourished mice express significantly more arginase both *in vitro* and *in vivo*. We proposed that increased arginase-mediated L-arginine catabolism is a mechanism likely to contribute to more severe disease by promoting parasite growth. Further work into the impact of arginase-mediated L-arginine catabolism during PEM is warranted as this may result in the identification of novel therapeutic dietary interventions that might interfere with this pathway and therefore improve immune responses and resistance to infectious diseases.

## Electronic supplementary material

Additional file 1: **High protein diet.** (PDF 176 KB)

Additional file 2: **Low protein diet.** (PDF 131 KB)
