# Supplementary material for: Protein energy malnutrition increases arginase activity in monocytes and macrophages
Source: Nutr Metab (Lond). 2014 Oct 24;11:51. doi: 10.1186/1743-7075-11-51 (PMC4228191; doi:10.1186/1743-7075-11-51)
Supplement: Supplementary file 2 — Additional file 2: Low protein diet. (PDF 131 KB) [file 12986_2014_622_MOESM2_ESM.pdf]

Additional file 2: description of the low protein diet

Modification of TestDiet® AIN-93M w/ No Protein 5B6A

DESCRIPTION

Mod TestDiet® AIN-93M without protein.

Storage conditions are particularly critical to TestDiet® products, due to the absence of antioxidants or preservative agents. To provide maximum protection against possible changes during storage, store in a dry, cool location. Storage under refrigeration (2° C) is recommended. Maximum shelf life is six months. (If long term studies are involved, storing the diet at -20° C or colder may prolong shelf life.) Be certain to keep in air tight containers.

*Product Forms Available**	*Catalog #*
Meal, Irradiated	1813232

*Other Forms Available On Re

INGREDIENTS (%)

Corn Starch	60.5692
Dextrin	15.5000
Sucrose	10.0000
Powdered Cellulose	5.0000
Soybean Oil	4.0000
AIN 93M Mineral Mix	3.5000
AIN 93 Vitamin Mix	1.0000
Choline Bitartrate	0.2500
L-Cystine	0.1800
t-Butylhydroquinone	0.0008

FEEDING DIRECTIONS

Feed ad libitum. Plenty of fresh, clean water should be available at all times.

CAUTION:
Perishable - store properly upon receipt.
For laboratory animal use only; NOT for human consumption.

2/4/2010

NUTRITIONAL PROFILE [1]

Protein, %	***0.7***
Arginine, %	0.00
Histidine, %	0.00
Isoleucine, %	0.00
Leucine, %	0.00
Lysine, %	0.00
Methionine, %	0.00
Cystine, %	0.18
Phenylalanine, %	0.00
Tyrosine, %	0.00
Threonine, %	0.00
Tryptophan, %	0.00
Valine, %	0.00
Alanine, %	0.00
Aspartic Acid, %	0.00
Glutamic Acid, %	0.00
Glycine, %	0.00
Proline, %	0.00
Serine, %	0.00
Taurine, %	0.00
Fat, %	***4.1***
Cholesterol, ppm	0
Linoleic Acid, %	2.04
Linolenic Acid, %	0.31
Arachidonic Acid, %	0.00
Omega-3 Fatty Acids, %	0.31
Total Saturated Fatty A	0.60
Total Monounsaturated Fatty Acids, %	0.88
Polyunsaturated Fatty Acids, %	2.16
Fiber (max), %	***5.1***
Carbohydrates, %	***87.0***
Energy (kcal/g) [2]	***3.85***

From:	kcal	%
Protein	0.029	0.8
Fat (ether extract)	0.371	9.6
Carbohydrates	3.482	89.7

Minerals	
Calcium, %	0.50
Phosphorus, %	0.20
Phosphorus (available), %	0.00
Potassium, %	0.36
Magnesium, %	0.05
Sodium, %	0.14
Chloride, %	0.20
Fluorine, ppm	1.0
Iron, ppm	35
Zinc, ppm	30
Manganese, ppm	11
Copper, ppm	6.0
Cobalt, ppm	0.0
Iodine, ppm	0.21
Chromium, ppm	1.0
Molybdenum, ppm	0.14
Selenium, ppm	0.17
Vitamins	
Vitamin A, IU/g	4.0
Vitamin D-3 (added), IU/g	1.0
Vitamin E, IU/kg	78.8
Vitamin K (as menadione), ppm	0.29
Thiamin Hydrochloride, ppm	6.0
Riboflavin, ppm	6.0
Niacin, ppm	30
Pantothenic Acid, ppm	15
Folic Acid, ppm	2.0
Pyridoxine, ppm	5.8
Biotin, ppm	0.2
Vitamin B-12, mcg/kg	25
Choline Chloride, ppm	1,250
Ascorbic Acid, ppm	0.0

1. Formulation based on calculated values from the latest ingredient analysis information. Since nutrient composition of natural ingredients varies and some nutrient loss will occur due to manufacturing processes, analysis will differ accordingly. Nutrients expressed as percent of ration on an As-Fed basis except where otherwise indicated.
2. Energy (kcal/gm) - Sum of decimal fractions of protein, fat and carbohydrate x 4,9,4 kcal/gm respectively.

ISO 9001:2000 CERTIFIED

TestDiet
www.testdiet.com
